# Dynamic Augmented Reality Cues for Telementoring in Minimally Invasive Surgeries: Scoping Review

**DOI:** 10.2196/63939

**Published:** 2025-02-03

**Authors:** Hawa Hamza, Omar M Aboumarzouk, Abdulla Al-Ansari, Nikhil V Navkar

**Affiliations:** 1 Department of Surgery Hamad Medical Corporation Doha Qatar

**Keywords:** minimally invasive surgery, surgeons, laparoscopic, telementoring, teleproctoring, telemedicine, augmented reality, dynamic visual cue, technologies, robotics, patient outcomes, communication, scoping review, PRISMA

## Abstract

**Background:**

Remote surgeons use telementoring technologies to provide real-time guidance during minimally invasive surgeries (MIS). Such technologies are continuously improving with the integration of dynamic augmented reality (AR) cues. This includes virtual overlays of hand gestures, pointers, and surgical tools onto the operating surgeon’s view. The operating surgeon comprehends this augmented information to operate on the patient. Thus, understanding these dynamic AR cues (either during surgical training or live surgery) is crucial.

**Objective:**

In this paper, we aimed to review the existing telementoring technologies that use dynamic AR cues during MIS. This review describes the MIS (including surgery type, specialty, procedure, and clinical trial), the telementoring system, the dynamic AR cues generated by these systems, and evaluation of the technology in terms of technical aspects, user perceptions, skills gained, and patient outcomes.

**Methods:**

A scoping review was conducted using PubMed, Web of Science, Scopus, IEEE Xplore, and ACM Digital Library databases. The search terms included “telementoring,” “minimally invasive surgery,” and “augmented reality” without restrictions imposed on the publication year. Articles covering telementoring using dynamic AR cues during MIS, including laparoscopic and robot-assisted, were identified.

**Results:**

A total of 21 articles were included and categorized based on type of surgery, the telementoring technology used, and evaluation of the technology. Most of the articles reported on laparoscopic suturing performed using synthetic phantoms. Hand gestures and surgical tools were the most frequently used dynamic AR cues (10 articles on each cue), while the mentors and mentees primarily consisted of experienced surgeons and medical students. The studies assessing the telementoring technologies were either descriptive (7 articles) or analytical (14 articles) where it was compared against no cue, prerecorded visual cue, in-person guidance, audio cue, or static AR cue. Outcomes were measured mostly using skills gained (13 articles) and user perception about the telementoring system.

**Conclusions:**

In general, telementoring using dynamic AR cues resulted in positive outcomes during MIS. In some cases, they were considered on par with conventional methods such as audio cues and in-person guidance. Further randomized controlled trials are required to objectively assess its clinical benefits.

## Introduction

One of the telemedicine technologies that is gaining considerable interest is telementoring, during which an expert medical professional (mentor) can provide real-time instructions to a novice (mentee) even if they are remotely located [1]. A growing application of telementoring technologies is seen in the field of surgery as a result of rising demands for specialized surgical expertise coupled with limited training opportunities [2]. Minimally invasive surgery (MIS) is one such specialized surgical field, which has emerged as the standard practice [3]. During MIS, which includes laparoscopic and robot-assisted procedures, surgeons use long, thin surgical instruments inserted through small incisions on the body. The operative view is captured through a camera scope and displayed on a screen. MIS is increasingly adopted due to the benefits it provides to patients, in terms of significant reduction in pain, surgical site infection, scarring, and recovery time as compared with the highly invasive open approach [4]. Despite these pronounced advantages, its widespread implementation is hindered due to the challenges in adequate skills acquisition. Surgeons performing MIS have an indirect view of the operative view through the screen projecting the live camera scope feed and experience a longer learning curve as compared with open surgery [5]. Hence, specialized training is needed to develop sufficient expertise. While basic MIS skills are learned outside the operating room, intraoperative training is vital for attaining proficiency [5]. Unlike open surgeries where the expert surgeon can easily point at critical structures with their hands over the operative view, mentors must rely on verbal instructions during MIS, which are prone to communication errors [6]. In some cases, the mentor must take complete control of the instruments from the mentee to show the required surgical technique. While the dual surgeon console in robot-assisted surgeries facilitates such takeovers by the mentor, the cost can be prohibitively high. Furthermore, many countries experience challenges in implementing MIS due to inadequate training programs and high costs associated with bringing in expert surgeons [7]. Telementoring technologies that allow an expert surgeon to efficiently guide the operating surgeon play a major role in addressing this gap [8].

Implementing telementoring technologies during MIS is relatively easier, as compared with open surgeries, since a live view of the operating field is already captured through the camera scopes [9,10]. During conventional telementoring, video captured from the operating room is transmitted in real time to an expert mentor at the remote location. Verbal instructions (audio cues) from the mentor are transferred to the mentee [11]. The mentor can also play surgical videos (prerecorded visual cues) to guide the mentee on a particular step [12]. However, such cues may not be sufficient for intraoperative instructions [6]. To overcome the communication challenges posed by verbal instructions, expert surgeons need a way to point at anatomical structures on the screen displaying the operative view to the mentee [13]. For this purpose, the mentor may use augmented reality (AR), which involves the use of computer-generated virtual objects that are superimposed on the mentee’s view of the operative field [14]. The AR cues may be rendered to the mentee on a visualization screen, or the surgeon’s console with stereoscopic vision during robot-assisted surgery. In some cases, external AR devices such as Microsoft HoloLens head-mounted display (HMD) or iPad tablets may also be used [15]. The cues could be in the form of 2-dimensional (2D) annotations (static AR cues) demonstrating an incision line or highlighting a specific anatomy [16,17]. Such forms of telementoring have been applied extensively for MIS demonstrating improved communication and performance [18]. Nonetheless, static AR cues are limited while compared with in-person guidance as complex tool-tissue interactions may not be accurately depicted [19,20].

Telementoring can be enhanced with the use of dynamic AR cues to effectively communicate surgical steps. As opposed to static AR cues, which remain stationary on the mentee’s field of view, dynamic AR cues move in real time under the mentor’s control [21]. These virtual cues can be in the form of hand gestures, pointers, or surgical tools. They are often 3-dimensional (3D) in nature, providing depth perception as the mentor guides the mentee through surgical sub steps. In cases where the mentor and mentee have similar macro skills, such as proficiency in critical anatomy and movement of surgical tools, these dynamic cues can aid the mentee’s development of micro skills related to a particular unfamiliar surgical technique [22]. Dynamic AR cues are not only useful for telementoring when the mentor is situated in a remote location, but can also be used to enhance in-person guidance during MIS where the mentor and mentee are in the same operating room [23].

Several reviews related to telementoring in health care have been previously published; however, they did not focus on applications for surgery in specific [15,24]. Other reviews covering surgical applications of telemedicine and telementoring have been presented [1,11,25-30]. These articles largely covered implementations of telementoring systems where video feed from the mentee is sent for audio feedback from the remote mentor. Some of them did not focus on MIS and explored applications in open surgery as well [11,29,30]. Additional review articles focused on specific use of telementoring for certain specialties such as head and neck [31], robotic [32], and urology [33]. With respect to MIS, Nickel et al [23] provided a concise clinical summary, whereas others reported on the use of audio and static AR cues [16,17,19,34,35]. While these reviews offer a comprehensive understanding of telementoring during MIS, they do not highlight the developments in the use of dynamic AR cues. To the best of our knowledge, there has not yet been a systematic examination specifically on the use of dynamic AR cues used by remote mentors during telementoring in MIS. Such a synthesis providing an overview of the different types of dynamic AR cues as well as the surgical procedures, clinical settings, and factors used for testing is needed. This will help guide future research and developments on telementoring in MIS and ensure that they are relevant and practical.

Through this scoping review we aim to provide a comprehensive summary of dynamic AR cues used for telementoring during MIS. A survey of the various types of surgeries, participants, dynamic AR cues, and evaluation of the telementoring technology is presented to guide future developments on telementoring during MIS. We aim to address the following research questions (RQs), which are yet to be answered by review articles published in the field:

- RQ1: What are the MIS specialties and procedures where dynamic AR cues have been used for telementoring? What are the clinical trials or settings they were tested on?
- RQ2: What are the different types of dynamic AR cues used for MIS telementoring? What information does the mentor convey to the mentee using these cues?
- RQ3: What factors are used to evaluate the use of dynamic AR cues during MIS telementoring? What are the common outcomes reported?

## Methods

### Overview

The review was conducted based on the PRISMA-ScR (Preferred Reporting Items for Systematic reviews and Meta-Analyses extension for Scoping Reviews) guidelines [36].

### Eligibility Criteria

To be considered eligible for the review, articles must satisfy the following criteria (Textbox 1).

**Eligibility criteria for scoping review.**

**Inclusion criteria**

- Original article.
- Reporting telementoring technologies using dynamic augmented reality cue.
- Reporting telementoring technologies during minimally invasive surgeries (including, endoscopic, laparoscopic, and robot-assisted).
- Published in English.

**Exclusion criteria**

- Articles on nonsurgical applications.
- Open surgery (ie, not minimally invasive surgeries).
- Microsurgery.
- Studies without telementoring technologies.
- Reports of telementoring without the use of augmented reality.
- Telementoring using static augmented reality cues (annotations) only.
- Articles without enough information (such as editorials and comments).
- Review articles.

### Search

A thorough search of scientific databases including PubMed, Web of Science, and Scopus was conducted. Other databases such as “IEEE *Xplore*” and “ACM Digital Library” covering technical fields were also included. The search terms comprised of, but were not restricted to, “telementoring,” “teleproctoring,” “augmented reality,” “overlay,” and “minimally invasive surgery.” There were no restrictions imposed on the year of publication. The latest search was conducted on March 11, 2024. The strategy used for searching in PubMed is provided in Multimedia Appendix 1. Additional records were found through citation searches and scanning review articles.

### Study Selection

Through the searches, a total of 460 scientific records were identified. Duplicate removal, title and abstract and full-text screening were done using the Rayyan web app [37]. Two reviewers [HH] and [NN] were independently responsible for title and abstract and full-text screening process. Resolution of disagreements on eligibility of an article was done through consensus and discussion with a third reviewer [OA] when necessary.

### Data Extraction

Data items extracted from the articles included year of publication, type of surgery, surgical specialty and procedure, phase of clinical trial, number of participants and their experience level, telementoring technology, type of dynamic AR cue, comparator (if applicable), factor measured, and outcome of the study. The data extraction form with the variables to be extracted from each eligible article were jointly developed by 2 reviewers [HH] and [NN] for this scoping review. The reviewers were independently responsible for extracting the data from each study. Resolution of disagreements on results of the data extraction was achieved through discussion with a third reviewer [OA] when necessary.

### Quality Assessment

Methodological quality assessment of the included studies was conducted based on the Medical Education Research Study Quality Instrument (MERSQI) [38]. The tool is designed to appraise the quality of medical education research, which was found to be appropriate for the topic addressed by this scoping review. The quality assessment tool was not used as an exclusion criterion, but instead, it was used after the screening process to gauge the quality and reliability of the articles reporting the use of dynamic AR cues during minimally invasive surgical telementoring [39]. Each article was assessed according to study design, type of data obtained, evaluation instrument used, analysis of the data, and outcomes measured to give a score out of 18. A higher score given to an article indicates a superior study design. Similar to the study selection and data extraction process, 2 reviewers [HH] and [NN] were responsible for quality assessment of the included studies, while disagreements were resolved through discussions with a third reviewer [OA] when necessary.

### Data Synthesis

The data extracted from the articles were presented using a descriptive table with information on surgery, participants (performing the procedure), dynamic AR cues, comparator, outcomes measured, and primary findings of the study. Study characteristics such as surgical procedure, clinical trial, type of dynamic AR cue, and publication year were visually summarized as counts. A meta-analysis was not conducted since a wide variety of outcomes measured, clinical setting, and type of participants was observed.

## Results

### Overview

**Figure 1:**
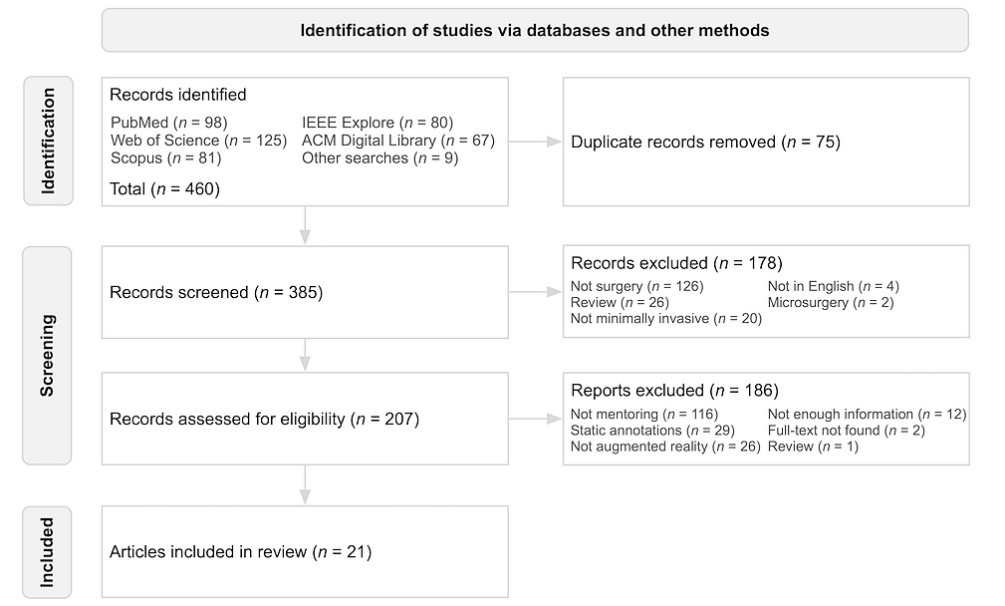
Flowchart depicting identification, screening, and inclusion of articles on the use of dynamic augmented reality cues during minimally invasive surgical telementoring.

A total of 21 articles reporting on the use of dynamic AR cues during telementoring in MIS were included in the review (Figure 1). An average MERSQI score of 12.9 was observed in the included articles. Table 1 presents a summary of the included articles. The table elements are described in detail in the following sections. They include details about the MIS procedures, the various dynamic AR cues used for telementoring, and evaluation of the technologies and their outcomes.

**Table 1:** Studies reporting dynamic augmented reality cues during minimally invasive surgery telementoring.

Study	MERSQI^a^ score	Minimally invasive surgery				Mentor and mentee		Intervention			Evaluation	
		Type^b^	Procedure	Clinical trial			Technology		Dynamic AR^c^ cue	Comparator		Outcome
Cizmic et al [6]	15.5	L	Cholecystectomy	Animal (cadaver)	Doctor trained by surgeon (n=1); medical students (n=40)		iSurgeon		Hand gesture	Audio cue		Skills: improved GOALS^d^, OSATS^e^ scores and fewer complications, no difference in operating time
Felinska et al [18]	14.5	L	Cholecystectomy; basic tasks	Animal (cadaver), virtual simulator, synthetic phantom	Mentor (n=1); medical students (n=40)		iSurgeon		Hand gesture	Audio cue		Skills: improved participant gaze behavior, lower number of errors, higher OSATS score in iSurgeon group
Huettl et al [40]	16	L	Cholecystectomy	Human (live)	Mentors (n=13); surgical trainees (n=15)		HoloPointer		Pointer	Audio cue		Perceptions, skills: reduced corrections, improved subjective performance, favorable user ratings; no difference in GOALS score, CVS^f^, and operating time
Long et al [41]	10	R	Prostatectomy; suturing, basic task	Human (live), synthetic phantom	Expert surgeon (n=1); trainee (n=1)		In-house developed system		Hand gesture, surgical tool	—^g^		Technical: latency within standard acceptable latency (330 ms)
Shabir et al [42]	12.5	L	Suturing	Synthetic phantom	Mentors (n=2); mentees (n=12)		In-house developed system		Surgical tool	In-person guidance		Skills: longer operating time observed, but comparable performance
Shabir et al [20]	11.5	L	Salpingectomy	Virtual simulator	Mentor (n=1); mentees (n=12)		In-house developed system		Surgical tool	In-person guidance		Skills: lower complication percentage during learning stage; lowered operating time during testing stage
Shabir et al [43]	12.5	L	Suturing	Synthetic phantom	Mentor (n=1); mentees (n=18)		In-house developed system		Surgical tool	Prerecorded visual cue, in-person guidance		Skills: lower error count, no difference in operating time
Lowry et al [44]	15.5	L	Suturing, basic task	Synthetic phantom	Mentor (n=1); premedical, medical students (n=30)		MVR^h^ HelpLightning platform		Surgical tool	No cue, in-person guidance		Skills: improvement compared to group who received no cue, similar FLS^i^ skills among telementored and in-person guided groups
Shabir et al [22]	10	L	Basic task	Synthetic phantom	Mentor (n=1); mentee (n=6)		In-house developed system		Surgical tool	—		Technical: latency of 260 ms observed to send information from mentee to mentor and 132 ms vice versa
Wild et al [13]	15.5	L	Cholecystectomy	Animal (cadaver)	Expert surgeon (n=1); medical students (n=60)		iSurgeon		Hand gesture	Audio cue		Perceptions, skills: faster operating time, lower error rate, improved GOALS and OSATS scores, lower complications, and reduced workload (NASA-TLX^j^)
Youssef et al [45]	9	R	Prostatectomy	Human (live)	Expert urology mentors (n=2); senior urology fellows (n=2)		Proximie		Hand gesture	—		Perceptions: favorable ratings provided by mentors and mentees
El-Asmar et al [46]	16	R	Aquablation for benign prostatic obstruction	Human (live)	Mentors (n=3); surgeons (n=not specified)		Proximie		Hand gesture	In-person guidance		Patient outcome: no difference in operating time, drop in hemoglobin, urinary retention, and complications; increased anesthesia use and cauterization under telementor guidance
Heinrich et al [47]	14.5	L	Cholecystectomy	Virtual simulator	Expert surgeon (n=1); surgical trainees (n=10)		HoloPointer		Pointer	Audio cue		Skills: improvement in economy of movement, error rate, and performance
Shabir et al [48]	11	L, R	Suturing	Synthetic phantom	Mentor (n=1); mentee (n=1)		In-house developed system		Surgical tool	—		Technical: latency of 1.56 s to transfer information from operating room to remote area and 0.089 s vice versa
Feng et al [49]	13.5	L	Cholecystectomy	Synthetic phantom	Fellow trained by surgeon (n=1); residents (n=6)		Virtual pointer		Pointer	Audio cue		Skills: improvement in turn frequency (number of turns per second)
Feng et al [50]	14.5	L	Cholecystectomy	Synthetic phantom	Fellow trained by surgeon (n=1); surgical trainees (n=7)		Virtual pointer		Pointer	Audio cue		Skills: improvement in economy of movement and subjective performance, no difference in errors and operating time
Jarc et al [21]	11	R	Suturing, basic task	Animal (live)	Expert surgeons (n=6); medical students, residents (n=7)		In-house developed system		Hand gesture, pointer, surgical tool	—		Perceptions: tool rated favorably by mentors and mentees
Kowalewski et al [51]	12.5	L	Suturing, basic task	Synthetic phantom	Surgeons, mentors (n=4); novice, intermediate, expert mentees (n=30)		iSurgeon		Hand gesture	—		Skills: positive correlation observed between iSurgeon parameters and OSATS score
Davis et al [52]	10	L	Ventriculostomy	Human (live)	Expert surgeon (n=1); expert surgeon (n=1)		VIPAR^k^		Hand gesture, pointer	—		Technical: latency of 237 ms in visual cue; successful implementation of VIPAR
Jarc et al [53]	11	R	Basic task	Animal (live)	Surgeons (n=26); surgical, nonsurgical trainees (n=26)		In-house developed system		Hand gesture, pointer, surgical tool	Static AR cue (2D telestration)		Perceptions: participants preferred 3D tools over 2D telestration
Vera et al [54]	14.5	L	Suturing	Synthetic phantom	Mentor (n=1); premedical, medical students (n=19)		ART^l^		Surgical tool	In-person guidance		Skills: steeper learning curve, faster operating time, fewer failed attempts in ART group

^a^MERSQI: Medical Education Research Study Quality Instrument.

^b^types: L: laparoscopic; R: robot-assisted.

^c^AR: Augmented Reality.

^d^GOALS: Global Operative Assessment of Laparoscopic Skills.

^e^OSATS: Objective Structured Assessments of Technical Skills.

^f^CVS: Critical view of safety.

^g^Not applicable.

^h^MVR: merged virtual reality.

^i^FLS: fundamentals of laparoscopic surgery.

^j^NASA-TLX: NASA (National Aeronautics and Space Administration) Task Load Index.

^k^VIPAR: virtual interactive presence and augmented reality.

^l^ART: augmented reality telementoring.

### MIS (Research Question 1)

**Figure 2:**
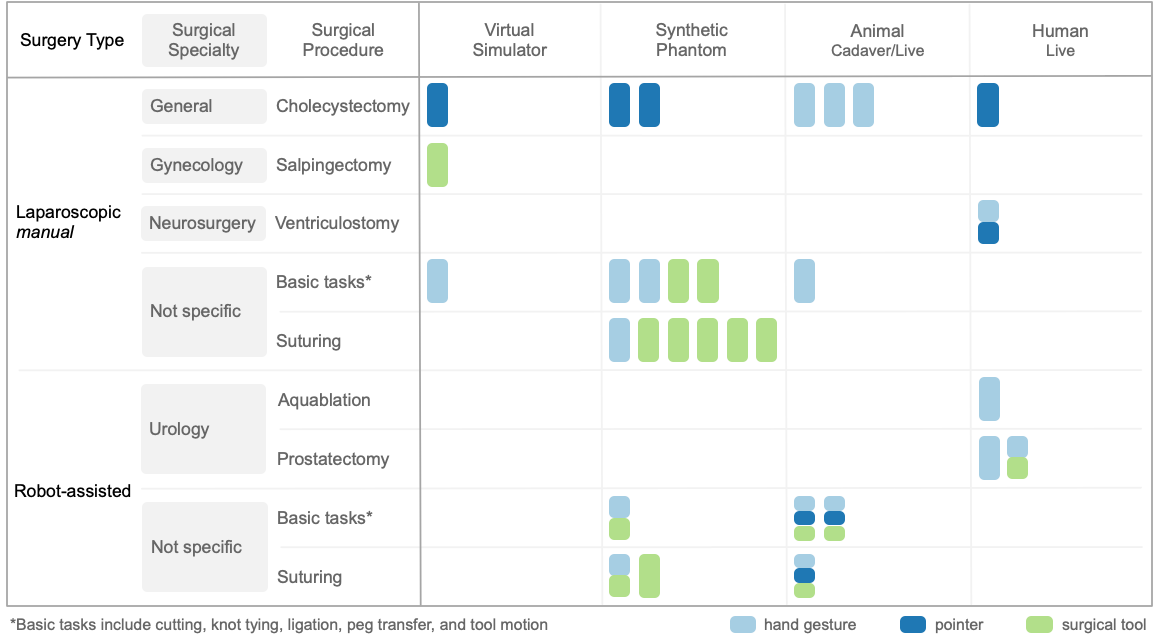
Depiction of surgical procedures and the corresponding clinical settings under which the telementoring technologies were tested.

Article on MIS included 2 categories, namely, manual or conventional laparoscopic (n=16 articles) and robot-assisted (n=6). These were subdivided based on surgical specialties, which consisted of general, gynecology, neurosurgery, urology, and nonspecific specialties. Figure 2 depicts the surgical procedures and the corresponding clinical settings under which the telementoring technologies were tested. Each vertical block represents an article using dynamic augmented reality cues. Articles using multiple dynamic cues are represented as segmented vertical blocks. The surgical procedures identified included cholecystectomy [6,13,18,40,47,49,50], salpingectomy [20], ventriculostomy [52], aquablation [46], and prostatectomy [41,45]. Nonspecific procedures consisted of basic tasks (such as cutting, knot tying, ligation, peg transfer, and tool motion) [18,21,22,41,44,51,53] as well as suturing [21,41-44,48,51,54]. The procedures were conducted under four different clinical settings: virtual simulator, synthetic phantom, animal (cadaver or live), or human (live). Virtual simulators present highly realistic computer-generated surgical procedures. However, they often have limited haptic feedback providing tactile sensation [55]. Synthetic phantoms consist of low or high-fidelity tissue models. Depending on the material it is made of, it may even provide realistic tissue interaction [56]. Animal models include euthanized cadavers or anesthetized live animals, while surgeries on real patients were categorized as human (live). All the 4 clinical settings were used for telementoring during cholecystectomy procedures. Maximum number of articles covered laparoscopic suturing on synthetic phantoms (n=6). A range of dynamic AR cues were used in articles covering robot-assisted basic tasks performed on animal models.

### Telementoring Technology (Research Question 2)

**Figure 3:**
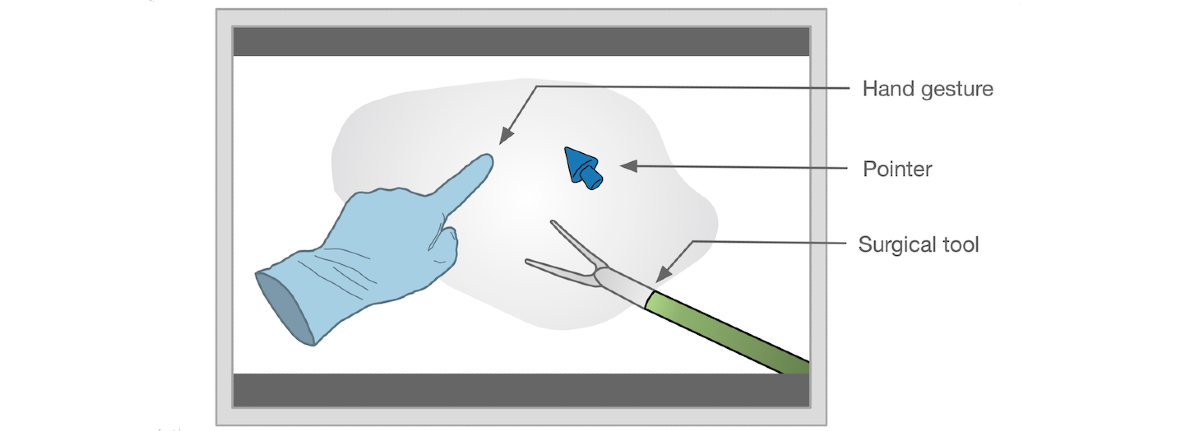
Various dynamic augmented reality cues overlayed on the mentee’s operative field.

**Figure 4:**
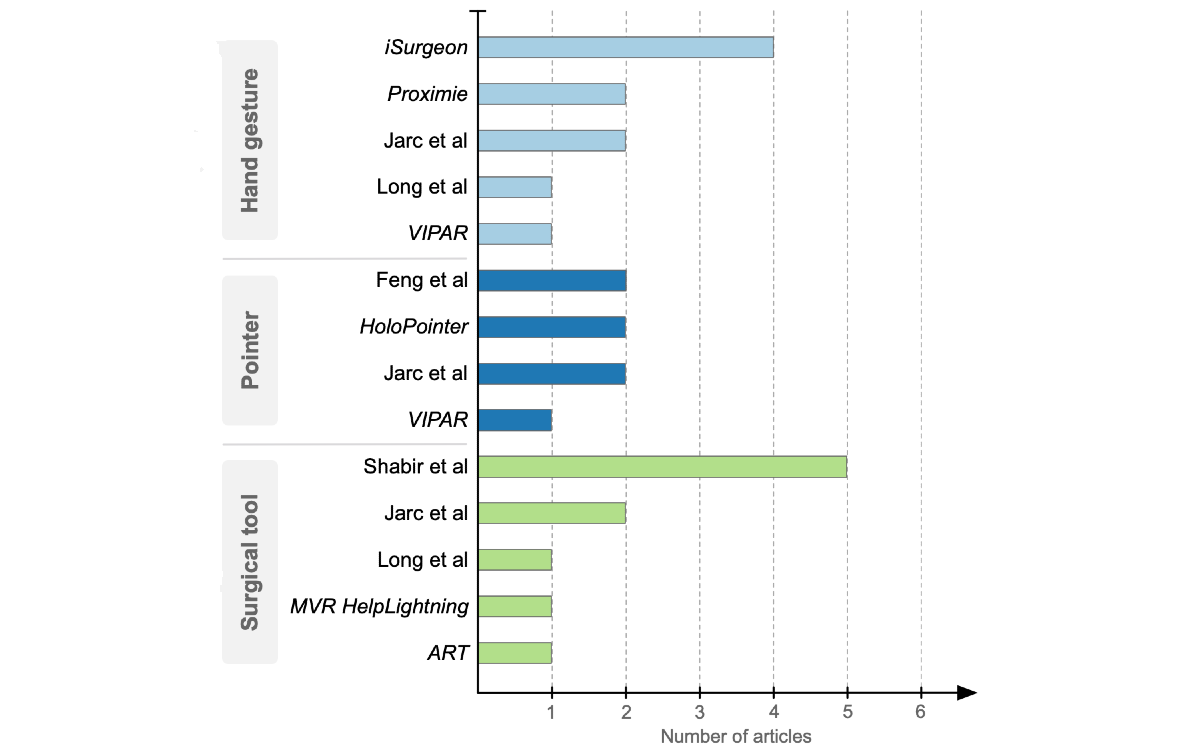
Number of articles reporting telementoring systems using dynamic augmented reality cues, specifically, hand gestures, pointers, and surgical tools [6,13,18,20-22,40-48,51-54]. ART: augmented reality telementoring; MVR: merged virtual reality; VIPAR: virtual interactive presence and augmented reality.

The mentors used various forms of dynamic AR cues that were overlaid on the mentee’s view of the operating field (Figure 3). These visual cues were divided into 3 types, hand gesture (displaying mentor’s hand position and orientation), pointer (in the form of dynamic annotations), and surgical tool (showing the type of tool, position, or required motion). As depicted in Figure 4 [6,13,18,20-22,41-46,48,51-54], most of the articles covered hand gestures and surgical tools (n=10 articles on each cue). The telementoring system developed by Jarc et al [21,53] used all the 3 types of dynamic AR cues. Some of the pros and cons of each cue are summarized in Table 2. The following paragraphs provide a detailed description of the three dynamic AR cues.

**Table 2:** Summary of common pros and cons of each dynamic augmented reality cue.

Dynamic augmented reality cue	Pros	Cons
Hand gesture	Natural and intuitive mode of communication [18]. Orientation and position can be conveyed [21]. Grasping tool actions can be mimicked using open and close gestures with fingers [53].	Mentee’s view of the operative field may get obstructed [43]. Complex tool tip movements may not be conveyed [48].
Pointer	Critical anatomical structures and dissection plane can be indicated [52]. Mentors can use hands-free interaction when pointing is enabled through head tracking [47].	Tool tip orientation may not be conveyed [48]. Complex instructions such as grasping and other tool movements may not be replicated [48]. Verbal clarification of the instruction may be required [53].
Surgical tool	Exact tool to be used, tool tip orientation, and position can be conveyed [44,54]. Required motion and open and close gestures of the tool can be conveyed [53]. Decreases the need for takeover of the surgery by the mentor [54].	Mentee’s view of the operative field may get obstructed [43]. Implementation of the technology may be challenging [22].

#### Hand Gesture

Hand gesture was one of the most used dynamic AR cues among the included articles. It enhances the mentoring experience with the ability to point at critical anatomical structures, demonstration of grasping gestures by opening and closing fingers, and depiction of orientation through hand rotation movements [13,53]. In the iSurgeon system, the mentor’s hand gestures were captured using an overhead depth video camera, which was then projected on to the mentee’s view of the operative field [6,13,18,51]. In this way, the mentor was able to support the mentee by communicating the dissection plain, target anatomy, and indicating the next steps of the procedures [6]. Similarly, the hand gesture overlays were also used in telementoring systems such as Proximie and Virtual Interactive Presence and Augmented Reality (VIPAR) [46,52]. In the VIPAR system, the video camera on iPad tablet devices were used to capture the mentee’s operative view as well as the mentor’s hand gestures. Both the video feeds were then combined allowing a transparent overlay of the mentor’s hand to appear in the mentee’s view [52]. The telementoring system developed by Jarc et al [21,53] integrated custom wireless input devices and commercial game controllers allowing the mentor to manipulate the orientation and position of a virtual hand appearing in the mentee’s operative view. The mentor was also able to indicate grasping gestures (open and close) using the input device. In this way, the mentee was able to understand and translate the hand gestures to orient, position, and manipulate the surgical tool accordingly. Consequently, the use of virtual hands for telementoring was rated as highly effective by both the mentors and mentees [53]. As reflected by its frequent usage observed in the included articles, hand gestures provide a natural and intuitive mode of communication for the mentor making it easier for the mentee to interpret and follow the instructions.

#### Pointer

The virtual pointer was used to indicate critical anatomy or a specific region in the mentee’s operative view. Frequency of its use as dynamic AR cue during telementoring was relatively lower compared with hand gestures and surgical tools among the included articles. Nevertheless, the use of pointers showed significant improvement in quality of instructions compared with verbal feedback alone [50]. The HoloPointer system used an AR HMD device (Microsoft HoloLens) to track head movements [40]. In doing so, the mentor was able to point at various regions of the operating field view in a hands-free manner [47]. In other studies, the mentors used hand movements, which were captured by a motion sensing device (Microsoft Kinect) to control the virtual pointer as described by Feng et al [49,50]. The VIPAR system, similar to its implementation for hand gestures, used an iPad tablet device to capture the pointing device used by the mentor and combine it with the mentee’s operative view [52]. The 3D pointers appearing on the mentee’s view were manipulated by the mentor using game controllers in the system presented by Jarc et al [21,53]. However, the use of 3D pointers received low ratings from the mentors. Overall, virtual pointers allowed the mentors to clarify verbal instructions by directing the mentee’s attention to a particular region of interest. However, it was not used as frequently, since the mentor is not able to convey precise instructions, such as tool tip orientation and motion.

#### Surgical Tool

Virtual surgical tools provided a close-to-reality visual cue during telementoring. It enhances the realism of the mentee’s training environment as the mentor can accurately convey the precise tool to be used, orientation and position of the tool tip, as well as the necessary movements (such as open and close gestures). Hence, the surgical tool as a dynamic AR cue was frequently used among the included studies. This was primarily done in 2 ways, by superimposing live video feed with real surgical tool from the mentor’s side on to the mentee’s view or by displaying 3D models of the surgical tool, which the mentor manipulates using various input devices. One of the earliest implementations, the augmented reality telementoring (ART) system, used a laparoscopic box simulator with an embedded camera at the remote mentor’s side. As the mentor maneuvered the real surgical tool, the live video feed was superimposed on the mentee’s view of the operative field. This resulted in improved understanding of instructions and quicker skills acquisition. The mentees also reported that the cue did not obstruct the operative view [54]. With respect to the use of 3D surgical tool models, Shabir et al [43,48] reported a telementoring system where the mentor did not use real tools and instead controlled the virtual tools appearing on the mentee’s view using a haptic device (Touch, 3D Systems). Similarly, Long et al [41] used an AR HMD device that tracked the mentor’s hand movements. These movements were then translated to manipulate the virtual tool instructing the mentee. Following the approaches for hand gestures and virtual pointers, Jarc et al [21,53] enabled the mentors to manipulate the ghost tools using custom input devices and game controllers. The virtual surgical tool models enabled the mentor to convey intricate tool wrist orientation information. Overall, surgical tools enhance the instructional experience by replicating complex motions during different surgery phases and overcoming communication barriers between the remote mentor and mentee. Despite the considerable benefits, the use of surgical tools as dynamic AR cues for telementoring in MIS is still in the early stages of clinical trial.

### Evaluation of Technology (Research Question 3)

The telementoring technologies using dynamic AR cues were evaluated with participants having varying expertise in MIS. Majority of the studies had experienced surgeons as mentors [13,21,41,45,47,51-53], whereas medical students [6,13,18,21,44,54] and surgical trainees [40,47,50,53] frequently formed the mentee group. The evaluation studies were identified as having either descriptive or analytical study designs. The descriptive studies (n=7 articles) presented the usability or feasibility of the proposed dynamic AR cue, whereas analytical studies (n=15 articles) used comparators to measure effectiveness. Such analytical studies compared outcomes of telementoring using dynamic AR cue against performance with no cue (independent performance without mentoring), prerecorded visual cue (video guidance), in-person guidance, audio cue, or static AR cue (2D telestration). Audio cue [6,13,18,40,47,49,50] and in-person guidance [20,42-44,46,54] were the most frequently used comparators.

The telementoring studies assessed the effectiveness of dynamic AR cues using a range of outcome measures. These were categorized broadly into factors based on technical evaluation and some of the items specified under the MERSQI outcomes domain [38]. Accordingly, the factors measured included technical, perceptions, skills, and patient outcomes. Technical factors (n=4 articles) included assessment of information latency between mentor and mentee locations [22,41,48,52]. Perceptions (n=5 articles) consisted of preferences, subjective performance assessment, user ratings, and workload measured using NASA (National Aeronautics and Space Administration) Task Load Index (NASA-TLX) [13,21,40,45,53]. Skills (n=13 articles) encompassed Critical View of Safety (CVS), economy of movement, fundamentals of laparoscopic surgery (FLS), gaze behavior, Global Operative Assessment of Laparoscopic Skills (GOALS), number of errors, operating time, Objective Structured Assessment of Technical Skills (OSATS), and turn frequency measuring communication skills [6,13,18,20,40,42-44,47,49-51,54]. Patient outcomes (n=1 article) included hemoglobin level, urinary retention, complications, and anesthesia use in a real surgery setting [46].

## Discussion

One of the aims of the scoping review was to present a comprehensive summary of the use of dynamic AR cues for telementoring during MIS. Through systematic searches of the scientific literature, it was observed that dynamic AR cues for telementoring were more frequently implemented for laparoscopic surgeries (as compared with robot-assisted surgeries). It was mainly used for teaching suturing skills on synthetic phantoms and remote mentoring during simulated cholecystectomy procedures. The mentors primarily consisted of expert surgeons, while medical students and surgical trainees dominated the mentee group. The use of hand gestures and surgical tools as dynamic AR cues were more frequent as compared with virtual pointers. Hand gestures and surgical tools allowed the mentor to convey information such as position, orientation, and tool tip motions to the mentee. To display the dynamic AR cues to the mentee, a small proportion of the studies used iPad tablets (n=1, 5%) Microsoft HoloLens (n=3, 14%), and the surgeon’s console with stereoscopic vision for robot-assisted surgery (n=4, 19%). However, most of the articles used a standard visualization screen (n=13, 62%) through which the mentee was able to see the AR cues. Among the included articles, at least 14 (67%) of them had a study design where the use of dynamic AR cue was compared with different modes of instruction with the most common comparators being audio cues and in-person guidance. The key factor used for evaluation was skills. The most frequently reported outcome measures were time taken for task completion and number of errors.

In general, the results of the included papers suggest that participants subjected to telementoring with dynamic AR cues experienced considerable beneficial effects when compared with groups receiving no cue, prerecorded visual cue, in-person guidance, audio cue, or static AR cue. More specifically, dynamic AR cues resulted in significant improvement of FLS score when compared with no cue [44] and lowered error count (*P*<.05) when compared with prerecorded visual cues [43]. While comparing against in-person guidance, some studies reported longer operating time [42], increased anesthesia use, and cauterization [46] with the use of dynamic AR cues. At the same time, few others noted a reduced complication percentage [20], lower operating time (*P*=.01), steeper learning curve, and fewer failed attempts [54]. Furthermore, several other studies found no significant difference in performance [42,44], number of errors [54], operating time [43,46], and clinical outcomes [46]. This indicates that telementoring using dynamic AR cues was similar to in-person guidance. While comparing with audio cues, some studies reported no significant difference in CVS, GOALS score [40], number of errors [50], and operating time [6,40,50]. However, the majority of studies demonstrated significant advantages of dynamic AR cues over audio cues. These were in terms of subjective performance [40,47,50], user ratings [40], NASA-TLX score (*P*<.022) [13], economy of movement [47,50], error rates [13,18,47], gaze behavior (*P*<.01) [18], GOALS score [6,13], operating time (*P*<.001) [13], OSATS score (*P*<.01) [6,13,18], turn frequency (*P*<.0001) [49], and complications [6,13]. These results show that dynamic AR cues were comparable, and significantly superior to telementoring with audio cues alone. In addition, a study by Jarc et al [53] noted that users preferred dynamic AR cues over static 2D telestrations. Overall, dynamic AR cues resulted in superior outcomes, and in some cases, they were considered on par with conventional methods such as in-person guidance and audio cues.

**Figure 5:**
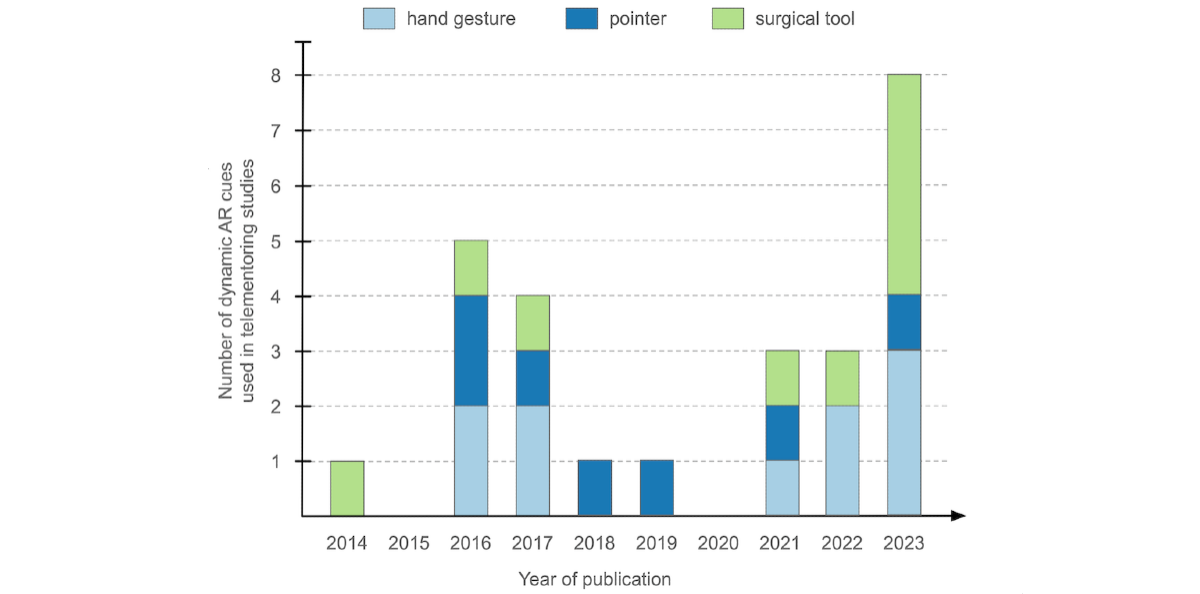
Number of articles using dynamic augmented reality cues in minimally invasive surgical telementoring.

The extent to which the above-mentioned findings may be used to determine the benefits of the technology is limited. This is because majority of the analytical studies with a comparator were conducted on synthetic phantoms, mostly assessing suturing [42-44,54]. Only 2 of the analytical studies were conducted in real patient settings comparing dynamic AR cue with audio during cholecystectomy [40] and in-person guidance during aquablation [46]. In addition, the outcome measures used to assess the different dynamic AR cues were not consistently used across the studies. The effectiveness of hand gestures was primarily assessed through OSATS, pointers through subjective performance, and surgical tools through latency and operating time. As a result, it is difficult to objectively compare between the different dynamic AR cues and determine the optimal one. Nevertheless, the articles published in the past few years reveal an upward trend in the use of hand gestures and surgical tools (Figure 5). This strongly indicates a rising interest in the use of dynamic AR cues for telementoring in MIS as well as the relevance of using hand gestures and surgical tools.

The implementation of telementoring using dynamic AR cues requires consideration of several technical factors. The Society of American Gastrointestinal and Endoscopic Surgeons (SAGES) issued a set of guidelines that telementoring systems are recommended to comply with. SAGES recommends that a minimum bandwidth of 40 megabits per second must be maintained. In addition, the latency (delay in sending mentee’s operative view to the remote mentor) must fall below 450 milliseconds [57]. One of the methods used to reduce latency is by encoding the video frames at the mentee’s location and decoding it at the mentor’s side. However, during this process, there is a risk of lowered video quality experienced by the mentor. To measure the extent of visual degradation experienced by the mentor, metrics such as structural similarity index measure (SSIM), peak signal-to-noise ratio (PSNR), and mean square error (MSE) are often used [58,59]. SSIM is considered more effective since it is more closely related to the visual quality perceived by the human eye, as compared with PSNR and MSE [22]. The delay experienced by the mentee in receiving mentor’s instructions can be reduced when point cloud data (depicting hand gestures or surgical tool) is used instead of video data. Harnessing 5G and future 6G capabilities that provide extremely low latencies can ensure little to no disruption [23]. While several studies included in the review reported information latency between mentor and mentee, they were mostly conducted on synthetic phantoms [22,41,48]. Further research presenting technical results during actual live MIS is imperative.

Ethical, safety, and legal concerns must be addressed while implementing telementoring systems. Informed patient consent must be ensured [23]. Every effort should be made to minimize cybersecurity risks and ensure compliance with guidelines on telemedicine issued by HIPAA (Health Insurance Portability and Accountability Act) [31]. The computer systems at both the mentor and mentee locations must be secure with monitoring schemes in place to detect and prevent potential breaches of sensitive patient data. To protect the data while being transferred between the mentor and mentee, appropriate data encryption and virtual private networks must be used [57]. In addition, a clearly outlined process for licensure and credentialing of surgeons is also required for remotely located mentors [8]. Generally, the mentee (who will be a competent operating surgeon) holds primary medical responsibility and is legally liable. Nonetheless, the SAGES states that during telementoring, the remote mentor retains shared responsibility for the patient [60]. Consequently, the mentor must hold the necessary medical license and surgical privileges in the region where the surgery is taking place. This becomes a barrier to adopting telementoring nationally in places like the United States and Canada as licensing is usually at the state or provincial level. However, this would not be a problem in the European Union (EU) where surgeons licensed under one EU member state can practice in other EU states as well [22]. Furthermore, licensing requirements would not be an issue when dynamic AR cues are used to enhance in-person guidance.

Telementoring using dynamic AR cues addresses the need for efficient ways to remotely guide an operating surgeon. This can be potentially replaced with telesurgery where the remote surgeon performs the surgery through robot-assisted surgical systems. While successful telesurgery cases were reported since the early 2000s, significant progress was made only in the recent years owing to the advances in surgical robot systems as well as telecommunication technologies [61]. However, several factors prevent widespread adoption of telesurgery. Although 5G networks are available, 3G or 4G is the standard in most countries leading to major concerns about latency. In addition, more research is needed to assess the cost-effectiveness of telesurgical services. A long-term sustainable approach must be explored for implementation and maintenance. Nonetheless, several cases of telesurgery for surgical specialties such as general, urology, and neurosurgery have been reported [62]. This provides hope for using remote technologies to reduce the global burden of death and disease that can be prevented or treated through surgical care.

This review is limited by the inclusion of articles published in English only, making it less comprehensive. A risk of bias assessment of the included studies was not carried out. A comparative analysis between the different dynamic AR cues was not conducted due to the variety in outcome measures reported. Instead, overall summary potentially guiding future research on dynamic AR cues used for MIS telementoring is presented.

Although further research is needed to establish its benefits over conventional mentoring, the use of dynamic AR cues in MIS telementoring proves to be promising. Future developments may focus on evaluating the technology through multi-institutional randomized controlled trials. In addition to measures of perceptions and skills, objective clinical outcomes that reliably assess the effectiveness of telementoring should also be evaluated.

## Appendix

Multimedia Appendix 1 Search strategy used for PubMed and other databases.

Multimedia Appendix 2 Preferred Reporting Items for Systematic reviews and Meta-Analyses extension for Scoping Reviews (PRISMA-ScR) checklist.

## Abbreviations

AR: augmented reality
ART: augmented reality telementoring
CVS: Critical View of Safety
EU: European union
FLS: fundamentals of laparoscopic surgery
GOALS: Global Operative Assessment of Laparoscopic Skills
HIPAA: Health Insurance Portability and Accountability Act
HMD: head-mounted display
MERSQI: Medical Education Research Study Quality Instrument
MIS: minimally invasive surgery
MSE: mean square error
NASA-TLX: NASA (National Aeronautics and Space Administration)Task Load Index
OSATS: Objective Structured Assessment of Technical Skills
PRISMA-ScR: Preferred Reporting Items for Systematic Reviews and Meta-Analyses extension for Scoping Reviews
PSNR: peak signal-to-noise ratio
RQ: research question
SAGES: Society of American Gastrointestinal and Endoscopic Surgeons
SSIM: structural similarity index measure
VIPAR: Virtual Interactive Presence and Augmented Reality
