# Supplementary material for: Dynamic Augmented Reality Cues for Telementoring in Minimally Invasive Surgeries: Scoping Review
Source: J Med Internet Res. 2025 Feb 3;27:e63939. doi: 10.2196/63939 (PMC11833267; doi:10.2196/63939)
Supplement: Multimedia Appendix 1 [file jmir_v27i1e63939_app1.docx]

**Supplementary Content 1**

Search strategy used for PubMed

| **#** | **Search Query** | **Results** |
| --- | --- | --- |
| 1 | "Telemedicine"[Mesh] OR “remote train*” OR “remote ment*” OR “telementoring” OR “teleproctoring” | 47,492 |
| 2 | "Augmented Reality"[Mesh] OR "Data Display"[Mesh] OR “overlay*” OR “annotation*” | 137,146 |
| 3 | "Minimally Invasive Surgical Procedures"[Mesh] OR "Laparoscopy"[Mesh] OR "Robotic Surgical Procedures"[Mesh] OR "Surgery, Computer-Assisted"[Mesh] OR (“minimally invasive” AND “investigational procedure”) | 641,999 |
| 4 | ("Telemedicine"[Mesh] OR “remote train*” OR “remote ment*” OR “telementor*” OR “teleproctor*” OR “telestrat*” OR “telepresence” OR “telecollaborat*”) AND ("Augmented Reality"[Mesh] OR "Data Display"[Mesh] OR “overlay*” OR “annotat*” OR “augment*”) AND ("Minimally Invasive Surgical Procedures"[Mesh] OR "Laparoscopy"[Mesh] OR "Robotic Surgical Procedures"[Mesh] OR "Surgery, Computer-Assisted"[Mesh] OR (“minimally invasive” AND “investigational procedure”)) | 98 |

Search strategy used for other databases

| **Database** | **Search Query** | **Results** |
| --- | --- | --- |
| Web of Science | ALL=((telemedicine OR remote train* OR remote ment* OR telementor* OR teleproctor* OR telestrat* OR telepresence OR telecollaborat*) AND (augmented reality OR data display OR overlay* OR annotate* OR augment*) AND (minimally invasive surg* OR laparoscop* OR robot* surg* OR computer-assisted surgery OR minimally invasive investigational procedure)) | 125 |
| Scopus | ( TITLE-ABS-KEY ( telemedicine OR "remote train*" OR "remote ment*" OR telementor* OR teleproctor* OR telestrat* OR telepresence OR telecollaborat* ) AND TITLE-ABS-KEY ( "augmented reality" OR "data display" OR overlay* OR annotat* ) AND TITLE-ABS-KEY ( "minimally invasive surg*" OR laparoscop* OR "robot* surg*" OR "computer-assisted surgery" OR "minimally invasive investigational procedure" ) ) | 81 |
| IEEE *Xplore* | ("All Metadata":telemedicine OR "All Metadata":remote train* OR "All Metadata":remote ment* OR "All Metadata":telementor* OR "All Metadata":teleproctor* OR "All Metadata":telestrat* OR "All Metadata":telepresence OR "All Metadata":telecollaborat*) AND ("All Metadata":augmented reality OR "All Metadata":data display OR "All Metadata":overlay* OR "All Metadata":annotate* OR "All Metadata":augment*) AND ("All Metadata":minimally invasive surg* OR "All Metadata":laparoscop* OR "All Metadata":robot* surg* OR "All Metadata":computer-assisted surgery OR "All Metadata":minimally invasive investigational procedure) | 80 |
| ACM Digital Library | [[All: telemedicine] OR [All: "remote train*"] OR [All: "remote ment*"] OR [All: telementor*] OR [All: teleproctor*] OR [All: telestrat*] OR [All: telepresence] OR [All: telecollaborat*]] AND [[All: "augmented reality"] OR [All: "data display"] OR [All: overlay*] OR [All: annotate*] OR [All: augment*]] AND [[All: "minimally invasive surg*"] OR [All: laparoscop*] OR [All: "robot* surg*"] OR [All: "computer-assisted surgery"] OR [All: "minimally invasive investigational procedure"]] | 67 |
